# Assessment of wasting and associated factors among under five children of Wukro town, Tigray regional, North Ethiopia: a cross sectional study

**DOI:** 10.11604/pamj.2019.33.330.18808

**Published:** 2019-08-30

**Authors:** Tesfay Tsegay Gebru, Gdiom Gebreheat Abady, Fisaha Gebretsadkan Teklu, Yohannes Ashebir Tesfamichael, Muzayene Tilahun Bitow, Kidanemaryam Berhe Tekola, Mulu Gebretsadik Weldemariam, Guesh Welu Gebreslassie, Haftom Tesfay Gebremedhin, Hagos Mehari Mezgebo, Senait Gebreslasie Gebremeskel, Mekonnen Haftom Goytom

**Affiliations:** 1 Department of Nursing, College of Medicine and Health Sciences, Adigrat University, Adigrat, Tigray, Ethiopia; 2 Department of Public Health, College of Medicine and Health Sciences, Adigrat University, Adigrat, Tigray, Ethiopia; 3 Department of Biomedical sciences, College of medicine and Health Sciences, Adigrat University, Adigrat-Tigray, Ethiopia; 4 Department of Midwifery, College of Medicine and Health Sciences, Adigrat University, Adigrat-Tigray, Ethiopia; 5 Department of Psychiatry, College of Medicine and Health science, Adigrat University, Adigrat, Tigray, Ethiopia

**Keywords:** Malnutrition, anthropometry, wasting, Wukro, Tigray, Ethiopia

## Abstract

**Introduction:**

Globally, 50 million children under 5 were wasted; of which 16 million were severely wasted. A severely wasted child is at a nine times higher risk of dying. To prevent this problem, it is necessary to determine the magnitude and factors associated with childhood wasting. In Ethiopia specifically Wukro town, Tigray regional state there is no clear information regarding under five wasting. Therefore, the study assessed the prevalence and associated factors of wasting among under five children in Wukro town, North Ethiopia. Objective: to assess the prevalence of wasting and associated factors among under five children of Wukro town, Tigray, North Ethiopia, 2017/2018.

**Methods:**

Community based cross-sectional study design with a single population proportion formula was used with a total sample size of 400 children. Wukro town has three kebele, two kebelle were included in the study through simple random sampling method. There was proportional allocation of subjects to each kebelle and final study subject was selected using systematic method. In case there were more than one child in the household one child was selected randomly. The data were collected by face to face interview and measuring of weight and height after the instrument was pre-tested. The anthropometric results were entered in to Emergency nutritional assessment (ENA) to calculate Z-Score. The collected data and result of Z-score were entered in to Statistical package for social science (SPSS) version 20. Finally, results were presented in texts, graphs and tables.

**Results:**

A total of 394 under five children were participated in this study, which gave a response rate of 98.5%. The respondents were females 222 (56.3%) and 106(26.95%) were in the age group of 12-23 month. The overall prevalence of wasting was 28 (7.2%). Out of this 14 (3.6%) were wasted and 14 (3.6%) were severely wasted. Under five children those, whose family does not live together were 3.086 times more likely to be wasted compared to under five children those, whose family live together (P=.038, OR=3.086, & 95% CI= (1.061, 8.970). Under five children those, whose mother did not taken family planning were 2.530 times more likely to be wasted compared to under five children those, whose mother take family planning (P=.038, OR=2.530, & 95% CI= (1.054, 6.074)).

**Conclusion:**

Significant numbers of mothers were not taken extra food during pregnancy and lactation. There was significant prevalence of wasting of under five children in the study area. Living condition of family and usage of family planning were associated with increased risk of wasting.

## Introduction

Nutrition is the provision of adequate energy and nutrients to the cells to perform their physiological function (of growth, reproduction, defense, and repair, etc) [1]. Malnutrition refers both to under nutrition and over nutrition, but the focus here is to under nutrition. Under-nutrition causes 175 deaths per 1000 children in low income countries compared to high income countries 6/1000 deaths. The nutritional status of women and children is particularly important, because it is through women and their off-spring that the pernicious effects of malnutrition are propagated to future generations. A malnourished mother is likely to give birth to a low birth-weight (LBW) baby susceptible to disease and premature death, which further undermines the economic development of the family and society, and continues the cycle of poverty and malnutrition. Malnutrition commonly affects all groups in a community, but infants and young children are the most vulnerable because of their high nutritional requirements for growth and development [2, 3]. Malnutrition due to primary lack of food and interplay of infections is known as primary malnutrition, which is responsible for most of the 112 million children suffering from moderate malnutrition in the developing world. Malnutrition occurring as a result of chronic diseases such as chronic kidney, liver or heart disease is known as secondary malnutrition. Although lack of food and repeated infections including diarrhea and pneumonia are the immediate, precipitating causes of malnutrition, the root causes are political in nature interlaced with issues of social and gender inequity particularly of income and education [4]. Causes of under nutrition that are being debated currently include growth faltering, low birth weight, maternal under nutrition, deficiencies of specific nutrients, diarrhoea, HIV infection and other infectious diseases, inadequate infant and child feeding practices, female time constraints, limited household income, limited agricultural production, food insecurity, environmental degradation, and urbanization. Optimal nutritional status results when children have access to affordable, diverse, nutrient-rich food; appropriate maternal and child-care practices; adequate health services; and a healthy environment including safe water, sanitation and good hygiene practices. These factors directly influence nutrient intake and the presence of disease. The interaction between under nutrition and infection creates a potentially lethal cycle of worsening illness and deteriorating nutritional status. Food, health and care are affected by social, economic and political factors [5, 6].

Globally in 2011, 52 million children under 5 years were wasted. The highest wasting prevalence is in South Asia, where approximately one in six children (16%) is wasted. In sub-Saharan Africa, nearly 1 in 10 children under the age of 5 (9%) were wasted. The number of wasted children in sub-Saharan Africa as a proportion of the world's total has increased over the same period of time. While a significant number of the world's 52 million wasted children live in countries where cyclical food insecurity and protracted crises exacerbate their vulnerability, the majority reside in countries not affected by emergencies [6]. Children who suffered from under nutrition are more likely to achieve lower educational levels than healthy children. The low education levels attained, often makes them less qualified for work, thus reducing their income-earning potential for non-manual work [7]. In 2014, the global wasting rate was 7.5%, approximately 1 out of every 13 children. Globally, 50 million children under 5 were wasted, of which 16 million were severely wasted [8]. The nutritional status of children is a reflection of their overall health. Under nutrition in childhood, is one of the main burdens of the health system and also affects the economic and socio-cultural status of society. Poverty and malnutrition play a crucial role in increasing morbidity and mortality, impairing cognitive development in children, and increasing common childhood infections. Acute malnutrition affects more than 50 million under-five children, causing 8% of child deaths globally each year. It is caused by poor maternal nutrition before and during pregnancy, inappropriate infant feeding practices, and repeated episodes of infections. A high percentage of illiterate mothers, limited access to safe drinking water, and poor hygiene and sanitation have also contributed to under nutrition and child morbidity in the country. Growing evidence suggests that 80% of childhood disease is related directly or indirectly to unsafe drinking water, inadequate sanitation and hygiene practices [9]. Under nutrition are clearly a major contributing factor to child mortality, disease and disability. A severely wasted child is at a nine times higher risk of dying. Brain and nervous system development begins early in pregnancy and is largely complete by the time the child reaches the age of 2 years. The timing, severity and duration of nutritional deficiencies during this period affect brain development in different ways. An estimated one third of deaths among children under age 5 are attributed to under nutrition. Under nutrition puts children at far greater risk of death and severe illness due to common childhood infections [6]. According to Ethiopian mini demographic and health survey, 2014, overall, 9% of Ethiopian children were wasted, and 3% are severely wasted. This coupled with inadequate feeding practices, may contribute to faltering nutritional status [10].

The study on the Cost of Hunger in Ethiopia has allowed us to quantify the negative impacts of child under nutrition in both social and economic terms. 28% of all child mortality in Ethiopia is associated with under nutrition. The annual costs associated with child under nutrition are estimated at Ethiopian birr (ETB) 55.5 billion, which is equivalent to 16.5% of GDP. For every additional case of child illness, both the health system and the families are faced with an additional economic cost. There were an estimated 378,591 additional annual cases of child mortality associated with child under nutrition, in the period from 2004 to 2009 [7]. The elimination of malnutrition in all its forms is an imperative for health, ethical, political, social and economic reasons, paying particular attention to the special needs of children, and women. Nutrition policies should promote a diversified, balanced and healthy diet at all stages of life. In particular, special attention should be given to the first 1,000 days, from the start of pregnancy to two years of age, pregnant and lactating women, women of reproductive age, and adolescent girls, by promoting and supporting adequate care and feeding practices, including exclusive breast feeding during the first six months, and continued breastfeeding until two years of age and beyond with appropriate complementary feeding [11]. In Wukro town, there is no clear information regarding the magnitude and factors that contribute to under five malnutrition. Therefore, the main aim of this paper was to assess the prevalence and associated factors of wasting among under five children and recommend necessary intervention methods to Tigray regional health bureau, Woreda Health Bureau and health care providers of the area. Likewise, the result of this study will be used as baseline information to Federal, regional and Woreda Health Office and professionals and local managers to prepare prevention method of wasting for the community, provide baseline information to researchers for further study in the area, supply important information for program managers and policy makers, provide pertinent information for curriculum designers to make necessary amendment and guide governmental and nongovernmental health organizations to focus and train health care providers.

## Methods

Community based cross-sectional study design was conducted from December, 2017-January, 2018. The study was conducted in eastern zone of Tigray, Wukro town. The town has three kebele administrations and had a total population of 30,210, of whom 14,056 men and 15,154 were women. A total of 9,383 households were counted in this town, resulting in an average of 3.22 persons to a household. The majority of the inhabitants practiced Ethiopian orthodox with 92.94%, while 6.03% of the populations were Muslim [12]. According to the information from the Woreda Health Bureau, the town had a total of 6,951 children under five. There were 2057 households with under five children in kebele 01, 590 households in kebele 02 and 1,860 households in kebele 03. Wukro town has two governmental health centers and one hospital. In addition to this there are 3 private health facilities and 6 pharmacy shop which provide health care services to the community. The study populations were all under five children of selected kebele of Wukro town administration. A sample of 400 under five children was included in the study. The sample size was determined using single population proportion formula. It was computed by considering 45.7% (p = 0.457) [10] prevalence of stunting in under five children, 95% confidence level to get maximum sample size and 5% margin of error. This resulted in 400 sample size after including 5% contingency for non-response rate.

Wukro town has three kebele of which two kebelle (01 and 03) were included in the study through lottery method. There was proportional allocation of subjects to each kebele and final study subject was selected after list of households with under five children were taken from health extension workers and systematic method was applied to collect information from each kebele based on the arrangement of houses. For Households with more than one child, one child was selected randomly. If the intended child and family were not available during data collection the next child was included in the sample and continues with the previous interval. Eligibility criteria was all under five children of selected kebele of Wukro town and available during data collection time with their care givers. Structured questionnaire was developed by the principal investigator after reviewing different related literatures with required modification based on outcome variables and their predictors. The questionnaire were prepared first in English then translated to the local language (Tigrigna). To check consistency of the translation; retranslation to English was done by other translator. Questionnaire was pre-tested on 5% of the same source population other than the sampled population. Based on the pre-test, questions were revised, edited, and those found to be unclear or confusing were modified. Finally, structured closed ended Tigrigna version questionnaire was used for data collection. Data was collected by face to face interview and measurement of anthropometry by using measuring instruments. Data collectors were 6 BSc Nurses and were supervised by 3 MSc nurses. They were trained for two days on the study instrument, data collection procedures and anthropometric measurements. Supervisors and principal investigator collect filled questionnaires every day and checked for consistencies and completeness.

### Definition of terms

**Wasting:** nutritional deficient state of recent onset related to sudden food deprivation or mal-absorption utilization of nutrients which results weight loss, weight-for-height below -2SD from the NCHS/WHO median value. Severe wastage was diagnosed if it was below -3 SD [13].

### Anthropometric measurement

**Height/length measurement:** body length of children age up to 23 months was measured without shoes and the heights were read to the nearest 0.1 cm by using a horizontal wooden length board with movable headpiece and the infant in recumbent position. However, height of children 24 months and above was measured using a vertical wooden height board by placing the child on the measuring board, and child standing upright in the middle of board. The child's head, shoulders, buttocks, knees and heels touching the board.

**Weight measurement:** weight was measured by electronic digital weight scale with minimum/lightly/clothing and no shoes. Calibration was done before weighing every child by setting it to zero. In case of children age below two years, the scale was allowed weighing of very young children through an automatic mother-child adjustment that was eliminated the mother's weight while she standing on the scale with her baby.

**Methods of data analysis:** data was checked for completeness and any incomplete information was excluded from entry. Anthropometric result was entered in to ENA (emergency nutritional assessment) to calculate Z-Score. The collected data and result of Z-score were entered in to SPSS version 20.0. Simple frequencies were run to see the overall distribution of the study subject with the variables under study. Level of interaction of independent variables (multicollinearity) was assessed through VIF and was found absence of interaction of independent variables. Multi-variable logistic regression analysis was run to identify the independent predictors of wasting of under five children. Adjusted odds ratio with 95% confidence interval was used to ascertain the association between dependent and independent variables. The level of significance was taken at α <0.05. Finally, result was presented in texts, and tables. Ethical clearance and approval was obtained from college of medicine and health science, Adigrat University. Official cooperation letter was written from Adigrat University research and community service directorate to Tigray regional health bureau. Then Tigray regional health bureau sent written cooperation letter to Wukro town administration health office. Official letter was obtained from Wukro town health office to each selected kebele. After explaining about the purpose, and the possible benefit of the study; written permission was obtained from each respondents. Confidentiality of the respondent was maintained throughout the study.

## Results

Participants' socio-demographic characteristics: a total of 394 under five children were participated in this study, which gave a response rate of 98.5%. The respondents were females 222 (56.3%) and 106 (26.95%) were in the age group of 12-23 month. About half 198 (50.3%) of the participants were between 2 and 3 in birth order and 194 (49.2%) had 4 to 5 house hold size. Orthodox Christianity was the dominant religion consisting of 341 (86.5%) followed by Muslim 53 (13.5%). Almost the entire participant were mothers and 393 (99.7%) were above the age of 18 years. Among all households 360 (91.4%) of head of households were father and 365 (92.6%) of the family live together. Almost 148 (37.6%) participants were live in their own house, but 239 (60.6%) live in house rent and the rest 7 (1.8%) live in their relatives house. Ninety eight percent of children were cared by their mother and 104 (26.4%) of households decided on use of money by mother and father jointly, 67 (17.0%) mainly by spouse, and 223 (56.6%) mainly by husband. Almost all children 393 (99.7%) were delivered at health facilities. Among the participants mothers 308 (78.2%) were housewife, 57 (14.5%) were merchant and the rest 29 (7.4 %) were government employed. Three hundred fifty five (90.1%) of families were lived together and the most common food of the family was injera with shiro wet in 100% of participants and almost 100% did not have food security the whole year (Table 1). Two hundred two (51.3%) of the children's gestational age were <37 completed weeks but 192 (48.7%) were between 37 and 42 weeks. Two hundred sixty (66%) of the children's birth weight were between 2500 and 4000 gram, 124 (31.5%) <2500 gram and the rest 10 (2.5%) were above 4000 gram. Almost all 390 (99%) of mothers gave the first milk (colostrum) to their child but only 4 (1%) of mothers squeezed out and throwed. A total of 387 (98.2%) participant children were not twin baby and 392 (99.5%) of children's were immunized according to age of the child. The main reason of under five children to stop taking breast feeding was mainly 167 (78%) because of age, 25 (12%) because of maternal health problem, 9 (4%) maternal pregnancy, 8 (4%) refusal by the child and the rest 4 (2%) was lack of milk by the mother. Hundred percent of the participant children did not have history of chronic illness (like childhood tuberculosis, diabetes myelitis, HIV/AIDS, heart problem and other chronic lung problems) and did not have history of hospitalization. Among the participant children only 89 (22.6%) were experienced diarrhea in the last 2 weeks before data collection, 55 (14%) had ARI (acute respiratory infection), 29 (7.4%) were febrile and 8 (2%) were developed measles. Nearly all 376 (95.4%) children had initiated breast feeding within one hour but 1 (0.3%) was initiated after 24 hour of delivery. Three hundred thirty four (84.8%) of children had started complementary feeding at 6 month but 19 (4.8%) was started after six month. Around Half, 213 (54.1%) of children had stopped taking breast feeding. Out of this 105 (49.1%) was taken for greater than two years but the rest 108(50.9%) for less than two years. Three hundred ten (78.7%) of children was received vitamin A in the last six month before data collection but 1 (0.3%) of the children received pre-lactal feeding immediately after delivery (Table 2).

**Table 1:** Socio-demographic characteristics of households of under five children in Wukro town, eastern zone, Tigray regional state, North Ethiopia, 2017/18

Variable		Frequency (N=394)	Percent (%)
Age of the child in month	0-6	37	9.4
	6-11	55	14.0
	12-23	106	26.9
	24-35	85	21.6
	36-47	77	19.5
	48-59	34	8.6
Sex of the child	Male	172	43.7
	Female	222	56.3
Birth Order of the child	1^st^	138	35.0
	2-3	198	50.3
	>=4	58	14.7
Household size	1	1	0.3
	2-3	127	32.2
	4-5	194	49.2
	6+	72	18.3
Religion	Orthodox	341	86.5
	Muslim	53	13.5
Mothers Education	Illiterate	46	11.7
	Primary	142	36.0
	Secondary and above	206	52.3
Marital status of mother	Single	16	4.1
	Married	367	93.1
	Divorced	11	2.8
Fathers education	Illiterate	27	6.9
	Primary	124	31.5
	Secondary and above	243	61.6
Mothers occupation	house wife	308	78.1
	Merchant	57	14.5
	government employed	29	7.4
Monthly income of family in Ethiopian Birr	<750	1	0.3
	750-1500	38	9.6
	>1500	355	90.1
Ethnicity of the family	Tigray	391	99.2
	Amhara	3	0.8

**Table 2:** Health care practice and morbidity of under five children in Wukro town, eastern zone, Tigray regional state, North Ethiopia, 2017/18

Variable		Frequency (N=394)	Percent (%)
Child ever taken to health facility	Yes	144	36.5
	No	250	63.5
Diarrhea in the last 2 week	Yes	89	22.6
	No	305	77.4
Acute respiratory infection(ARI) in the last 2 week	Yes	55	14.0
	No	339	86.0
Fever in the last 2 week	Yes	29	7.4
	No	365	92.6
Measles in the last 2 week	Yes	8	2.0
	No	386	98.0
Time of initiation of Breast feeding	Within one hour	376	95.4
	1-24 hour	17	4.3
	After 24 hour	1	.3
Is the child still taking breast feeding	Yes	181	45.9
	No	213	54.1
Duration of breast feeding	<24 month	108	50.7%
	>=24 month	105	49.3%
Initiation of complementary feeding	Before six month	8	2.0
	At 6 month	334	84.8
	>6 month	19	4.8
	Not introduced still now	33	8.4
Materials used for complementary feeding	Cup	148	41.0
	Bottle	69	19.1
	Spoon	143	39.6
	Hand	1	0.3
Immunization status of child	Not immunized	2	.5
	Immunized according to age	392	99.5
Vitamin A received in the last 6 month	Yes	310	78.7
	No	84	21.3
Child received pre-lactal food?	Yes	1	.3
	No	393	99.7
Preceding birth interval of baby	<=1 year	390	99.0
	1-2 year	4	1.0
Number of under five children	1	317	80.5
	>=2	77	19.5

Nighty four percent of under five children exclusively beast feed for 6 months but 8 (2%) were exclusively breast feed for less than six month. Among mothers who gave the response, 76 (19.3%) gave their first birth before 18 years. Two hundred eighty three (71.8%) and 368 (93.4%) of mothers were taken extra food during pregnancy and lactation respectively. Almost all 388 (98.5%) of mothers had ANC follow up, Out of this 206 (53.1%) mothers had >=4 times ANC follow up. A total of 303 (76.9%) of participant mothers used FP and Depo-Provera were used by 230 (75.9%) mothers. Fifty nine (15%) of respondent mothers were faced illness during pregnancy. In all households the sources of drinking water were tap water, and all households had nearby water source and latrine. More than half of households 279 (70.8%) had private pit/cement slab latrine and 258 (65.5%) of the latrine had washing facilities nearby. Most of the participants 313 (79.4%) used soap for hand washing after toileting. Almost all households 393 (99.7%) were used common pit waste disposal method and 392 (99.5%) participants wash hands before preparing food (Table 3). The overall prevalence of wasting was 28 (7.2%). Out of this 14 (3.6%) were wasted and 14 (3.6%) were severely wasted. Before bivariate and multivariate analysis multicollinearity diagnosis was assessed and absence of multicollinearity was satisfied. In bivariate analysis Mothers educational status, whether family live together, and whether mothers take family planning were associated with wasting. Variables which have P-value less than or equal to 0.25 in bivariate analysis were selected as a candidate for multivariate analysis. The multi-variable result showed that child's age, mothers education level, whether child still taking breast feeding, mother take extra food during pregnancy, and number of ANC follow up were not statistically associated with wasting of under five children but whether family live together, and mother's take family planning were significantly associated with wasting. That is Under five children those, whose family does not live together were 3.086 times more likely to be wasted compared to under five children those, whose family live together (Table 3). (P=.038, OR=3.086, & 95% CI= (1.061, 8.970). Under five children those, whose mother did not taken family planning were 2.530 times more likely to be wasted compared to under five children those, whose mother take family planning (Table 4). (P=.038, OR=2.530, & 95% CI= (1.054, 6.074).

**Table 3:** Hygienic condition of house hold of under five children in Wukro town, eastern zone, Tigray regional state, North Ethiopia, 2017/18

Variable		Frequency (N=394)	Percent (%)
Type of latrine	private pit/wooden slab	115	29.2
	private pit/cement slab	279	70.8
Presence of Washing facilities nearby latrine	Yes	258	65.5
	No	136	34.5
Use of soap for hand washing after toileting	Yes	313	79.4
	No	81	20.6
Type of waste disposal method	open field	1	0.3
	common pit	393	99.7
Care giver wash hand before preparing food	Yes	392	99.5
	No	2	0.5
Floor of the house	Earth floor	59	15
	Ceramics floor	335	85.0

**Table 4:** Factors associated with wasting in Wukro town, eastern zone, North Ethiopia, 2017/18

Variable	Nutritional status		COR (95% CI)	AOR (95% CI)	P-Value
	Normal, n (%)	Wasted, n (%)			
Child’s age	366(92.9%)	28(7.1%)	0.981(0.953, 1.009)	0.994(0.947, 1.044)	0.816
**Mothers Education**					
Illiterate	39(84.8%)	7(15.2%)	1	1	
Primary school	135(95.1%)	7(4.9%)	0.289(0.096, .874)	0.339(0.107, 1.074)	0.066
Secondary and above	192(93.2%)	14(6.8%)	0.406(0.154, 1.072)	0.341(0.114, 1.016)	0.053
**Family live together**					
Yes	333(93.4%)	22(6.6%)	1	1	
No	33(86.2%)	6(13.8%)	2.75(1.042, 7.267)	3.086(1.061, 8.970)	0.038*
**Is the child still taking breast feeding?**					
Yes	165(91.2%)	16(8.8%)	1	1	
No	201(94.4%)	12(5.6%)	0.616(0.283, 1.338)	0.802(0.205, 3.133)	0.750
**Mother take extra food during pregnancy**					
Yes	267(94.3%)	16(5.7%)	1	1	
No	99(89.2%)	12(10.8%)	2.023(0.924, 4.427)	0.672(0.286, 1.578)	0.362
**If yes, Number of ANC Follow up**					
<4 times	164(90.1%)	18(9.9%)	1	1	
>=4times	196(95.1%)	10(4.9%)	0.465(0.209, 1.035)	0.448(0.178, 1.124)	0.087
**Mother’s take family planning**					
Yes	287(94.7%)	16(5.3%)	1	1	
No	79(86.8%)	12(13.2%)	2.725(1.24, 5.996)	2.530(1.054, 6.074)	0.038*

* indicated the presence of association with the independent variable

## Discussion

The study result showed that 376(95.4%) of under five children were initiated breast feeding within one hour of delivery, 1 (0.3%) of child had received prelactal feeding, 94% received exclusive breast feeding until six month of age, 334 (84.8%) started complementary feeding at the age of six week, 283 (71.8%) and 368 (94.3%) of mothers take extra food during pregnancy and lactation respectively, 388 (98.5%) of mothers visited health facilities for ANC during pregnancy and 303 (76.9%) of mothers used family planning. Out of this 230 (75.9%) of mothers used depo-provera as family planning. This result were comparably higher than the study done in North Shewa, Oromia regional state 546 (66.6%) of children were initiated breastfeeding practice immediately after birth. 372 (45.4%) children were received prelactation of food. Children (770 (93.9%) were exclusively breastfeed until six months. About 453 (48.7%) of children started complementary feeding at the age of 6 months. 630 (77%) of mothers did not take extra food during pregnancy or lactation. About 685 (84) % of mothers visited health facilities for ANC during pregnancy. 672 (82%) mothers were used family planning and Depo-Provera 633 (77.2%) was used by majority of the mothers [11]. The difference might be due to difference in time duration and socio-demographic characteristics. The study result showed that the prevalence of wasting was 14 (3.6%) and 14 (3.6%) were severely wasted. Males were more wasted than females (18.6%, 12.6%) respectively). Only 1 (0.3%) of children was received prelactal feeding, and 94% of children were received exclusive breast feeding for 6 month. This result is comparatively different except the prevalence with the study conducted in Nzego district rural Tanzania which shows the prevalence of wasting was 6.5%. Girls were more wasted than boys (8.8% vs. 4.4%). About one third of children (33.5%) were given prelactal feeds. Only 22.9% of mothers practice exclusive breast feeding at least in the first three months of child's life [14]. This difference could be due to sociocultural difference of the two study area.

This study result is comparatively lower than the study conducted in Sudan Khartoum, that's the prevalence of sever wasting was 7.3%. Severe wasting was 11% among males while it was 4% among females [15]. This difference could be due to difference in socio-demographic characteristics and study period. The study result was also comparatively similar with study result of EDHS 2014 that indicates that, 9% of Ethiopian children were wasted, and 3% were severely wasted. Male children are slightly more likely to be wasted (10%) than female children (7%) [10]. This similarity could be due to similarity in socio-demographic characteristics. This study indicated that 76(19.3%) participant mothers gave their first birth before the age of 18 years. One hundred eleven (28.2%) and 26(6.6%) were not received extra food during pregnancy and lactation respectively. Under five children those, whose family does not live together were 3.086 times more likely to be wasted compared to under five children those, whose family live together (Table 4). (P=.038, OR=3.086, & 95% CI= (1.061, 8.970)). Under five children those, whose mother did not taken family planning were 2.530 times more likely to be wasted compared to under five children those, whose mother take family planning. (P=.038, OR=2.530, & 95% CI= (1.054, 6.074)). This study is relatively lower than the study done in Dollo Ado district, Somali region Ethiopia, 385(71.2%) of mothers gave first birth when they were younger than 18 years of age. Among those who had experienced at least one pregnancy, 392(72.5%) no extra food was taken from the usual time during pregnancy and lactation. Mother's education and prevalence of wasting were inversely related. Children who had ARI in the preceding two weeks of the survey were 1.96 times higher risk of being wasted compared to those who are not suffered with ARI(AOR=1.96 95% CI=1.20,3.18).

The odds of being wasted was 1.69 times higher among male children than female children (AOR=1.69 95% CI=1.16, 2.45). The risk of being wasted was 1.55 times higher among mother of children who consumed extra food during pregnancy than those mother of children who had not consumed extra food during pregnancy (AOR=1.55 95% CI=1.06, 2.26) [16]. This difference might be due to difference on socio-demographic characteristics of the study participants and living condition of the participants. Based on this study, almost all 393(99.7%) of children were delivered at health facilities but 1(0.3%) were delivered at home but 89(22.6%) of children were experienced diarrhea in the last 2 weeks of data collection, 55(14%) had experienced ARI, 29(7.4%) were febrile and 8(2%) were developed measles. Almost all, 392(99.5%) of children under five year were immunized according to age of the child and 310(78.7%) of children was received vitamin A in the last six month. This study is comparatively similar except place of delivery with the study conducted in North Shewa, Oromia regional state 652(79.5%) of children were delivered at home and 168(20.5%) children were delivered at health facilities. 212(25.9%) of children had diarrhea in last two weeks before study conducted and 55(6.5%), 48(5.9%) and 19(2.3%) of children had fever, ARI and measles, respectively. 785(95.7%) of children were immunized and 766(93.3%) of children were supplemented with vitamin A [17]. This similarity may be due to similarities on socio-demographic characteristics of participants.

## Conclusion

Based on the finding of this research significant numbers of mothers were not taken extra food during pregnancy and lactation. Significant number of households does not have washing facilities nearby latrine. There was significant prevalence of wasting of under five children in the study area. Living condition of family and usage of family planning were associated with increased risk of wasting. Therefore, Special emphasis should be provided feeding of pregnant and lactating mothers. All households should have washing facilities nearby latrine. As much as possible family should live together and use family planning methods. Intervention should focus on improving nutritional status of under-five children. Further research on consequence of wasting, and micronutrient deficiency is required.

### What is known about this topic

- The prevalence of wasting in rural Tanzania was 6.5%. Girls were more wasted than boys (8.8% vs. 4.4%);
- The prevalence of sever wasting in Sudan Khartoum, was 7.3%. Severe wasting was 11% among males while it was 4% among females;
- According to mini EDHS 2014, 9% of Ethiopian children were wasted and 3% were severely wasted. Male children are slightly more likely to be wasted (10%) than female children.

### What this study adds

- The prevalence of wasting in rural Tanzania was 6.5%. Girls were more wasted than boys (8.8% vs. 4.4%);
- The prevalence of sever wasting in Sudan Khartoum, was 7.3%. Severe wasting was 11% among males while it was 4% among females;
- According to mini EDHS 2014, 9% of Ethiopian children were wasted and 3% were severely wasted. Male children are slightly more likely to be wasted (10%) than female children.

## Competing interests

The authors declare no competing interests.
